# Robotic Rollator Assistance for Sit-to-Stand Transfers in Older Adults with Mobility Challenges: Within-Participant Feasibility Study

**DOI:** 10.2196/101303

**Published:** 2026-11-11

**Authors:** Anas Mahdi, Theresa Buchner, Christian Werner, Yue Hu, Katja Mombaur

**Affiliations:** 1 University of Waterloo, 200 University Avenue West, Waterloo, ON, N2L 3G1, Canada, 1 519-888-4567; 2 Geriatric Center, Medical Faculty Heidelberg, Heidelberg University, Heidelberg, Germany; 3 Karlsruhe Institute of Technology, Karlsruhe, Germany; 4 Systems Design Engineering, University of Waterloo, Waterloo, ON, Canada

**Keywords:** sit-to-stand, assistive robotics, smart walker, older adults, biomechanics, user experience

## Abstract

**Background:**

Robotic mobility aids with sit-to-stand (STS) support may help older adults reduce physical effort during a demanding, fall-prone daily task.

**Objective:**

This paper reports a feasibility evaluation of the “SkyWalker” robotic walker, which provides powered STS assistance via actuated lower handles. The 2 trajectories (linear and S-shape) were selected to examine how assistance profile shape influences feasibility, biomechanics, and user experience.

**Methods:**

Seven older adult patients with mobility challenges were enrolled from a rehabilitation hospital. One withdrew during the robot familiarization (unable to stand up) without attempting any study trial, and 6 completers (4 women, 2 men; aged 70‐84 years) form the analyzed sample. Participants completed 3 within-subject conditions: SkyWalker with the linear trajectory, SkyWalker with the S-shape trajectory, and a conventional rollator. We quantified STS task performance, perceived exertion Borg Category-Ratio 10 scale (Borg CR10), kinematics (center-of-gravity [CoG] height), kinetics (vertical ground-reaction force [Fz], normalized to body weight), and technology acceptance using an adapted Tele-healthcare Satisfaction Questionnaire–Wearable Technology (TSQ-WT). Participant-level data are the primary presentation. Condition effects were tested with exact nonparametric tests.

**Results:**

Under linear assistance, 27 of 30 repetitions (90%) were successful (mean 4.50, SD 0.84; 4 of 6 participants at 5/5) and perceived exertion was the lowest of the 3 conditions (Borg CR10 mean 1.50, SD 1.38; lower than the rollator in 3 of 6 participants, unchanged in 2, higher in 1). Under S-shape assistance, 21 of 30 repetitions (70%) were successful (mean 3.50, SD 1.97; 3 of 6 at 5/5), with exertion comparable to the conventional rollator (mean 2.50, SD 2.43 vs mean 2.33, SD 1.63). All 6 completers achieved 5 of 5 repetitions with the conventional rollator, so robotic assistance did not increase the number of successful transfers in this cohort. Exact tests showed no significant condition effect on exertion (Friedman *χ*^2^_2_=2.1; exact *P*=.40; Kendall W=0.18) or success (Friedman *χ*^2^_2_=5.0, exact *P*=.11; Kendall W=0.42). Group CoG curves showed earlier, more monotonic elevation for linear, and per-participant Fz showed greater between-participant variability in load-transfer timing under S-shape. Technology acceptance was heterogeneous (TSQ-WT totals 22‐77 of 80; 4 of 6 participants at 64 or higher).

**Conclusions:**

Powered STS assistance was feasible for most assisted transfers: 27 of 30 repetitions (90%) succeeded under the linear trajectory, which also produced the lowest mean perceived effort of the 3 conditions. The S-shape trajectory was more demanding, producing more partial completions and no effort reduction relative to the conventional rollator. In this first evaluation of SkyWalker with its intended users, the assistance profile, not powered assistance itself, is the variable to prioritize in further testing, and the results, which motivate trajectory personalization and brief familiarization, are informing the design of the next SkyWalker generation.

## Introduction

### Motivation: Sit-to-Stand as a High-Value Target for Mobility Support

Population aging is accompanied by a growing prevalence of mobility limitations that restrict independence and increase care needs. Mobility limitations are strongly associated with adverse health outcomes (eg, higher fall risk, health care usage, and reduced quality of life [QoL]), and they frequently co-occur with conditions that reduce strength, balance, and confidence in everyday movement [1]. Within this broader landscape, the sit-to-stand (STS) transition, rising from a chair to upright stance, is a particularly consequential activity of daily living. STS is performed repeatedly throughout the day (eg, from chairs, toilets, and during bedside transfers), and difficulty completing STS can become an early and persistent bottleneck for independent living: when standing up becomes unreliable, walking, toileting, and basic household participation are immediately threatened.

From a biomechanical perspective, STS is not simply “standing up”; it is a coordinated whole-body maneuver that requires (1) generating forward and upward momentum, (2) transferring support from a large base (chair + feet) to a smaller base (feet only), and (3) stabilizing the body in upright stance [2,3]. Classic analyses of chair-rise biomechanics describe characteristic phases (eg, flexion momentum, momentum transfer, extension, and stabilization), with the momentum-transfer portion often being the most balance-critical due to rapid changes in support and center-of-mass (CoM) motion [2]. Age and impairment-related reductions in lower-limb strength and postural control can therefore force compensatory strategies (greater trunk flexion, reliance on upper-limb push, slower or segmented transitions) that may maintain task completion yet increase fatigue, instability, and fear of falling [4,5].

Clinically, STS performance is widely treated as an indicator of lower-extremity function and balance capacity. Biomechanics and gerontology studies have shown that chair-rise performance differs systematically with age and functional status [4], and that functionally impaired older adults often adopt distinct strategies that reflect limited reserves and altered coordination [5]. These observations motivate STS not only as a measurement target but also as a design target: if an assistive device can make STS more feasible and less effortful while preserving stable and repeatable weight transfer, it could deliver immediate functional benefit (more successful transfers and less exertion) and potentially support safer daily routines. Accordingly, this paper focuses on walker-based assistance during STS, with the rationale for this choice discussed next.

### Rationale for Rollator-Based Active STS Assistance

Walking aids, especially rollators, are among the most common mobility supports for older adults because they can provide balance assistance, allow weight-bearing through the upper limbs, and fit naturally into daily environments. In parallel, recent “smart” or instrumented rollators have expanded the role of walking aids from purely mechanical supports to systems that can sense user state, infer intent, and offer context-dependent assistance (eg, navigation support, fall-risk monitoring, task-specific stabilization) [6]. For STS specifically, many older adults already use a walker to pull or push themselves upward, even though conventional rollator use during STS can be mechanically suboptimal (eg, relying on arm strength and handle forces that may destabilize the device if brakes are not properly engaged). This makes STS a prime candidate for task-adaptive assistance within a walker form factor: the device is already present in the environment and physically coupled to the user at the moment STS assistance is needed.

Active (powered) STS assistance embedded in a walker can, in principle, address two intertwined demands of STS: (1) raising the CoM (reducing required lower-limb extensor effort) and (2) managing balance during momentum transfer (shaping the trajectory of support and encouraging a stable forward progression). However, achieving these benefits depends critically on how assistance is delivered. Assistance that is too abrupt, poorly timed, or misaligned with the user’s intended strategy can increase coordination demands and reduce perceived safety, particularly for users with limited strength reserves or heightened concern about falling. This is where trajectory design and control policy matter: even with the same hardware, different assistance profiles may produce meaningfully different biomechanical and experiential outcomes.

### Related Work: STS-Assist Rollators, Mechanisms, and Control Approaches

Research on STS supporting rollators spans a range of mechanical designs and control philosophies. Early work demonstrated feasibility of embedding a standing-assistance mechanism into a walker frame and coordinating force/position control to support a natural rise. For example, Chugo et al [7] proposed a rehabilitation walker with a compact parallel-link mechanism and a controller that switches assistance modes based on the user’s posture, aiming to provide “natural assistance” informed by nursing practice. Related follow-up work expanded the system-level concept toward standing and walking assistance with a single device [8]. Building on these ideas, Chugo et al [9] also proposed motion control for continuous assistance across standing, walking, and seating operations, emphasizing mode switching and practical usability in daily-life sequences.

Beyond posture-based mode switching, other control approaches explicitly estimate user state/load to adapt assistance online. Morita et al proposed standing motion assistance on a robotic walker based on real-time estimation of the patient’s load, and used that estimate to select force/position control modes intended to preserve the user’s remaining physical capability [10]. More recent design efforts have also targeted domestic practicality and cost, for example, by combining minimal actuation (eg, gas-spring assisted lifting) with stabilization strategies and validating the concept in users with low levels of care [11]. Recent reviews and systems work continue to expand this design space, including intelligent walking-support robots that explicitly target the STS transition [12], proxy-based control of an assistive walker for intentional STS transfer [13], lightweight assistive rollators evaluated with older users [14], and dedicated STS-assist devices for older adults [15].

In parallel, several studies have explored alternative assistive kinematics and walking-aid form factors for STS. Bulea and Triolo [16] presented a vertical lift walker designed to reduce upper- and lower-extremity force demands during sit-to-stand while remaining usable as a mobility aid, demonstrated in a prototype evaluation with an individual with spinal cord injury. Earlier walking-support concepts also addressed STS transfer assistance by guiding the motion through the walking-aid interface (eg, robotic walking support systems for sit-to-stand assistance) [17].

Alongside mechanism and control innovation, several studies explicitly ground assistance in human movement principles. Geravand et al [18] modeled the STS transfer to enable biologically inspired robot assistance, with the goal of coordinating robot behavior with human motion in an intuitive and safe way. Complementary biomechanical evidence highlights the balance demands around seat-off (momentum transfer), reporting similar seat-off balance measures in older versus younger adults despite slower execution in the older group [19]. Optimization-based work has further examined how external support forces should be applied to best assist geriatric STS transfers [20], and has been extended to optimize the physical design of assistive devices for STS [21].

Despite this progress, the STS-assist walker literature remains comparatively small relative to other assistive domains (eg, gait exoskeletons) and is methodologically heterogeneous. Importantly, evaluation in the intended population has begun to emerge: Werner et al [22] evaluated an STS assistance system integrated into a smart walker in older adults with motor impairments, reporting improved STS success rates and high user satisfaction. More broadly, systematic reviews of robotic rollators emphasize that evaluations vary widely in target users, outcome measures, and study settings, which makes it difficult to generalize device benefits or identify which design features drive user-relevant improvements [23,24].

### Remaining Gaps Toward Real-World Adoption of STS-Assist Rollators

For STS-assist rollators aimed at older adults with mobility challenges, 3 gaps repeatedly limit translation from prototype promise to practical adoption.

#### Gap 1: Evaluation with the Intended Users in Realistic Contexts

Many assistive robotics studies demonstrate proof-of-concept performance in controlled settings, often using convenience samples. However, older adults with mobility challenges are a heterogeneous population with varied strength levels, balance strategies, and prior experience using mobility aids. Evidence gathered with healthy participants may therefore miss coordination difficulties, safety concerns, or training needs that emerge in clinical users [25,26]. Real-world deployment contexts such as geriatric hospitals, where users may already be fatigued, cautious, or recently injured, shape feasibility in ways that cannot be inferred from laboratory-only evaluations.

#### Gap 2: Linking Biomechanics to User Experience

Even when studies report performance improvements, they often do not explain *how* assistance reshapes movement strategy and loading, or how those biomechanical changes relate to perceived exertion and acceptance. Yet STS is biomechanically rich: assistance can alter CoM timing, foot loading progression, and the repeatability of weight transfer [2,3]. These characteristics are likely to influence perceived stability and effort, especially during momentum transfer. Without concurrent biomechanical and subjective data, it is difficult to determine whether a device reduces physical demand at the cost of increased coordination demand, or whether certain assistance profiles better support both mechanics and experience.

#### Gap 3: Acceptance Needs Explicit Measurement

Adoption of assistive technology depends on perceived usefulness, ease of use, trust/safety, and identity-related concerns (eg, stigma). Technology acceptance models emphasize that perceived usefulness and perceived ease of use drive intention to use, and that these determinants can be moderated by experience and context [27]. In mobility-impaired older adults, acceptance is additionally shaped by perceived safety, confidence, and fit with daily routines [28]. In social robotics and assistive robotics more broadly, scoping reviews similarly highlight that acceptability is multi-factorial and cannot be inferred from performance alone [29,30]. For STS-assist rollators, acceptance therefore needs to be measured explicitly and interpreted alongside task feasibility and mechanical effects.

### Aim and Research Questions

The aim of this study was to evaluate the feasibility of powered STS assistance delivered by the SkyWalker robotic rollator in older adult patients with mobility challenges in a geriatric-hospital setting, comparing 2 assistance trajectories (linear and S-shape) against a conventional rollator. This is the first evaluation of the SkyWalker with its intended end users. Our earlier work on the platform addressed device development and control rather than user outcomes [31]. We therefore combine in a single protocol the outcomes that govern adoption: task performance, perceived exertion, instrumented kinematics and kinetics of the transfer, and technology acceptance, each read alongside the participant’s baseline physical function and fall-related concern. We formulate the evaluation around 4 research questions (RQs) that are directly tied to measurable outcomes in our protocol:

1. RQ1 (Feasibility): Is active STS assistance with SkyWalker feasible for older adults with mobility challenges, as reflected by the ability to complete repeated STS trials safely?
2. RQ2 (Effort): Does SkyWalker STS assistance reduce perceived exertion during repeated STS compared with a conventional rollator condition?
3. RQ3 (Biomechanics): How do different assist trajectories (linear vs S-shape) influence STS biomechanics, specifically CoM elevation timing and vertical load transfer patterns, and do these profiles suggest differences in movement repeatability or coordination demand?
4. RQ4 (User experience): How do older adult users perceive the STS assistance in terms of benefit, usability, stigma/self-concept, and quality-of-life relevance, and how do these perceptions relate qualitatively to observed feasibility and biomechanics?

This study is a single-site within-participant feasibility study. Its contributions are (1) an evaluation with geriatric rehabilitation patients in a real clinical setting, (2) a within-subject comparison of assistance profiles (linear vs S-shape) against a conventional rollator, and (3) an integrated outcome set combining feasibility, perceived exertion, instrumented biomechanics, and user acceptance, addressing common evaluation fragmentation in robotic rollator studies [23].

## Methods

### SkyWalker Robot Platform

SkyWalker is a bilevel-handle powered rollator designed to support walking and to provide powered assistance during the STS transition. In this paper, we focus on the STS assistance function; walking support is not evaluated as an outcome. The device provides 2 sets of handles (Figure 1): fixed upper handles for post-STS standing/ambulation, and actuated lower handles that deliver the assist during STS. The lower handles execute a coordinated vertical lift with a gentle forward translation to support forward lean and safe CoG progression. For this study, 2 parameterized assist trajectories were used: a linear profile and an S-shaped profile.

**Figure 1.**
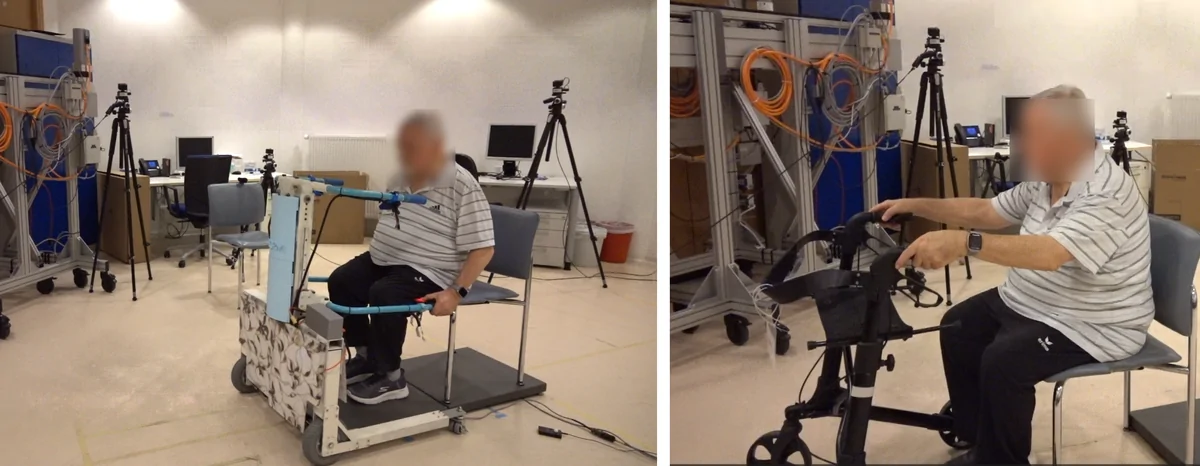
Experimental setup and assistive conditions during sit-to-stand trials. Left: participant performing a sit-to-stand trial using the SkyWalker assistive device under the supervised study protocol. Right: participant performing the corresponding trial using a conventional rollator. In the SkyWalker conditions, 2 force plates recorded ground-reaction forces (1 under the chair and 1 under the participant’s feet). The conventional-rollator condition was not instrumented with force plates. A Theia3D markerless motion-capture system recorded full-body kinematics in all conditions. Both panels illustrate the overall layout of the devices.

The actuated lower handles are position-controlled: each axis tracks a motion-profiled reference with continuous S-curve velocity shaping (bounded velocity, acceleration, and jerk) executed at the motor-controller level, and the coordinated 2-axis handle path during STS is generated by a kinematic trajectory generator. Assistance is therefore trajectory-based rather than force- or impedance-based. Handle start and end positions were set for each participant based on measured body height, while the trajectory execution time was fixed at 3 seconds for all participants and was identical for the linear and S-shape profiles, which differ in path shape rather than duration. Safety provisions during clinical testing included mechanical end stops on the actuated axes, software motion limits, overcurrent fuses that cut motor power on excessive current draw, and an operator emergency-stop button. A member of the study team (TB) supervised every repetition.

### Participants

Seven patients (age 70‐84 years; 5 women, 2 men) with mobility challenges were enrolled. One participant (woman, age 70; Clinical Frailty Scale [CFS] score 5, Short Physical Performance Battery [SPPB] score 4/12, Short Falls Efficacy Scale–International [Short FES-I] score 11/28, Mini-Mental State Examination [MMSE] score 28/30) withdrew during the initial “get to know the robot” familiarization (the study record notes that the patient was not able to stand up) and attempted no study trials, contributing no condition-level outcome data; thus, the analyzed sample comprised 6 participants (4 women and 2 men). The study took place at the rehabilitation wards of a geriatric hospital in Germany (Agaplesion Bethanien Heidelberg).

Inclusion criteria were age 65 years or older, ability to walk 4  meters with or without an assistive device, sufficient cognitive ability to follow study instructions, and self-reported difficulty performing the STS transition without external assistance. Examples of difficulty included requiring the use of the chair armrests or another support surface, needing physical assistance or close supervision, repeated attempts or prolonged time to stand, or feeling unstable during the rising phase. Exclusion criteria included cognitive impairment (MMSE score below 24 of 30) [32], as sufficient cognitive function was required to understand and follow the study instructions, interact safely with the device, and provide reliable self-reports on the questionnaires. All participants regularly used a walker or rollator for mobility. Notably, 3 of the 6 completers (and the participant who did not complete testing) could not perform a single chair rise without arm use in the SPPB chair-stand test, confirming that the sample represents the intended user group with genuine STS difficulty.

Cognitive status was screened using the MMSE (0‐30; higher =better cognition). Physical function was evaluated using the SPPB (0‐12; higher =better function) under standardized instructions, with established interpretive bands in older adults: approximately 10‐12 (near-normal), 6‐9 (moderate limitation), and less than 6 (marked limitation) [33]. Concern about falling was assessed using the 7-item Short FES-I (range 7‐28; higher =greater concern about falling) [34]. Notably, no participant reported being “very concerned” about falling in all situations; even those with higher FES-I scores identified only specific activities such as dressing or reaching that provoked concern rather than a generalized fear of movement. Fall history (number of falls in the previous year) was captured by patient self-report.

### Study Design and Procedure

Participants completed three within-subject conditions in randomized order (a script generated a random condition sequence for each participant): conventional rollator (no powered assistance), SkyWalker linear trajectory, and SkyWalker S-shape trajectory. For each condition, participants attempted up to 5 STS repetitions, with rest as needed between conditions; if a participant could not complete an STS attempt, the trial was stopped and counted as a failure for that repetition. A repetition was classified as a user-initiated abort when the participant released the handles, verbally requested to stop, or sat back down before reaching upright stance. All STS trials were performed from the same height-adjustable chair, with seat height adjusted to each participant’s body height. During the SkyWalker conditions, one force plate was positioned under the chair and one under the participant’s feet, whereas the conventional-rollator condition was performed without force plates. Rollator handle height was likewise adjusted per participant (86‐97 cm across the sample). Before recorded trials, the study team demonstrated hand placement and the handle motion. Participants then practiced assisted repetitions (approximately 3‐5) until they reported feeling comfortable and ready to begin the experiment.

1. No powered assistance: Participants used a standard wheeled walker (conventional rollator) for balance/support. The participant was seated in a stable chair and instructed to stand using the rollator as needed, without any powered assistance. This condition reflects a typical mobility aid used in daily life.
2. SkyWalker linear trajectory: Participants used the SkyWalker with STS assistance activated. During the rising phase, the SkyWalker’s lower handles moved in the horizontal and vertical directions simultaneously to support the lift, emphasizing a direct, coordinated upward assistance profile.
3. SkyWalker S-shape trajectory: Participants used the SkyWalker with STS assistance activated, but with an S-shape lower-handle trajectory. In this profile, the handles moved upward with a forward/curved component, resulting in a more complex handle motion pattern.

### Outcome Measures and Instruments

#### Task Performance

During each condition, we recorded the number of successful STS repetitions out of five. A repetition was counted as successful if the participant managed to stand up fully (upright posture) without physical assistance from another person and, in the case of the SkyWalker conditions, without aborting the device mid-lift. Time to complete 5 repetitions was recorded where possible but is not analyzed here because several participants could not complete all 5 in some conditions; we therefore focus on the count of successful stands (success rate) as a more universally applicable metric.

#### Perceived Exertion

After each condition, participants immediately rated their perceived exertion using the Borg Category-Ratio 10 scale (Borg CR10 scale; 0‐10 rating, where 0=“No effort at all,” 5=“Hard,” and 10=“Maximal effort”). This provided a subjective measure of how physically demanding the STS task felt under each condition.

#### Technology Acceptance

After completing the three conditions, participants completed a German-language adaptation of the Tele-healthcare Satisfaction Questionnaire–Wearable Technology (TSQ-WT) [35], adapted for the SkyWalker STS assistance system. The original TSQ-WT comprises 6 domains (benefit, usability, self-concept, privacy and loss of control, QoL, and wearing comfort) scored on a 5-point Likert scale from 0 (“not at all / completely disagree”) to 4 (“fully agree”), with higher values reflecting more positive evaluations. The instrument is designed to be adaptable to different systems; for this study, it was tailored to the STS assistance context by removing the domains that were not appropriate: “wearing comfort,” which targets body-worn devices, and “privacy and loss of control,” which focuses on long-term use. The resulting 20-item version evaluates user satisfaction and acceptance across 4 domains: benefit, usability, self-concept/stigma, and QoL. The same German-language adaptation, removing these 2 domains, has been used previously by our group to evaluate the STS assistance of a smart walker in older adults with motor impairments [22]. Negatively phrased items (items 2, 7, 8, 11, and 15) were reverse-coded so that, for all items, higher scores indicate a more positive attitude. Items are distributed unevenly across domains in the adapted instrument: benefit (7 items, maximum 28), usability (6 items, maximum 24), self-concept/stigma (4 items, maximum 16; higher =less stigma), and QoL (3 items, maximum 12). Domain scores were obtained by summing the items within each domain, and the total TSQ-WT score (range 0‐80) was obtained by summing all 20 items. The questionnaire was administered once, after exposure to both robotic trajectories. It therefore reflects acceptance of the SkyWalker system as a whole rather than of an individual trajectory, and it was not administered for the conventional rollator. The questionnaire was administered in German, using the German-language version of the TSQ-WT adopted from the HeiAge research project. No translation was performed within this study, and the English item wordings reported in Multimedia Appendix 1 are translations of the administered German items. To our knowledge, a formal comprehensive psychometric validation of the TSQ-WT, including validation of the German translation, has not been published. Its scores are therefore reported as descriptive indicators of acceptance rather than as validated acceptance measurements. The full list of items and their domain assignments is provided in Multimedia Appendix 1. The TSQ-WT has previously been used to assess wearable technology and robotic devices [22,36,37].

### Biomechanical Data Acquisition and Processing

#### Kinematics

Full-body kinematics were recorded with a markerless multicamera motion-capture system and processed with the Theia3D full-body model. The whole-body center of gravity (CoG) was taken from the model output (100 Hz), interpolated, and smoothed (Savitzky–Golay filter). Movement onset was detected with a sliding-window change-point rule on CoG height (rise of at least 2.5 cm between adjacent 0.5-s windows with confirming vertical velocity). For each participant and condition, repetitions were aligned at onset and averaged. Group curves are means of the per-participant mean curves resampled to a fixed per-condition analysis window (SkyWalker conditions: 1 s before onset to 4.5 s after onset; conventional rollator: onset to 2.0 s after onset, reflecting the shorter unassisted transfer). Each curve is displayed against movement percentage, defined as the percentage of the fixed analysis window rather than of each repetition’s own duration (Figure 2). Group curves include successful repetitions only and only participants with at least one successful repetition in the respective condition. Accordingly, the S-shape group curve comprises 5 participants (P3 completed no successful S-shape repetition).

**Figure 2.**
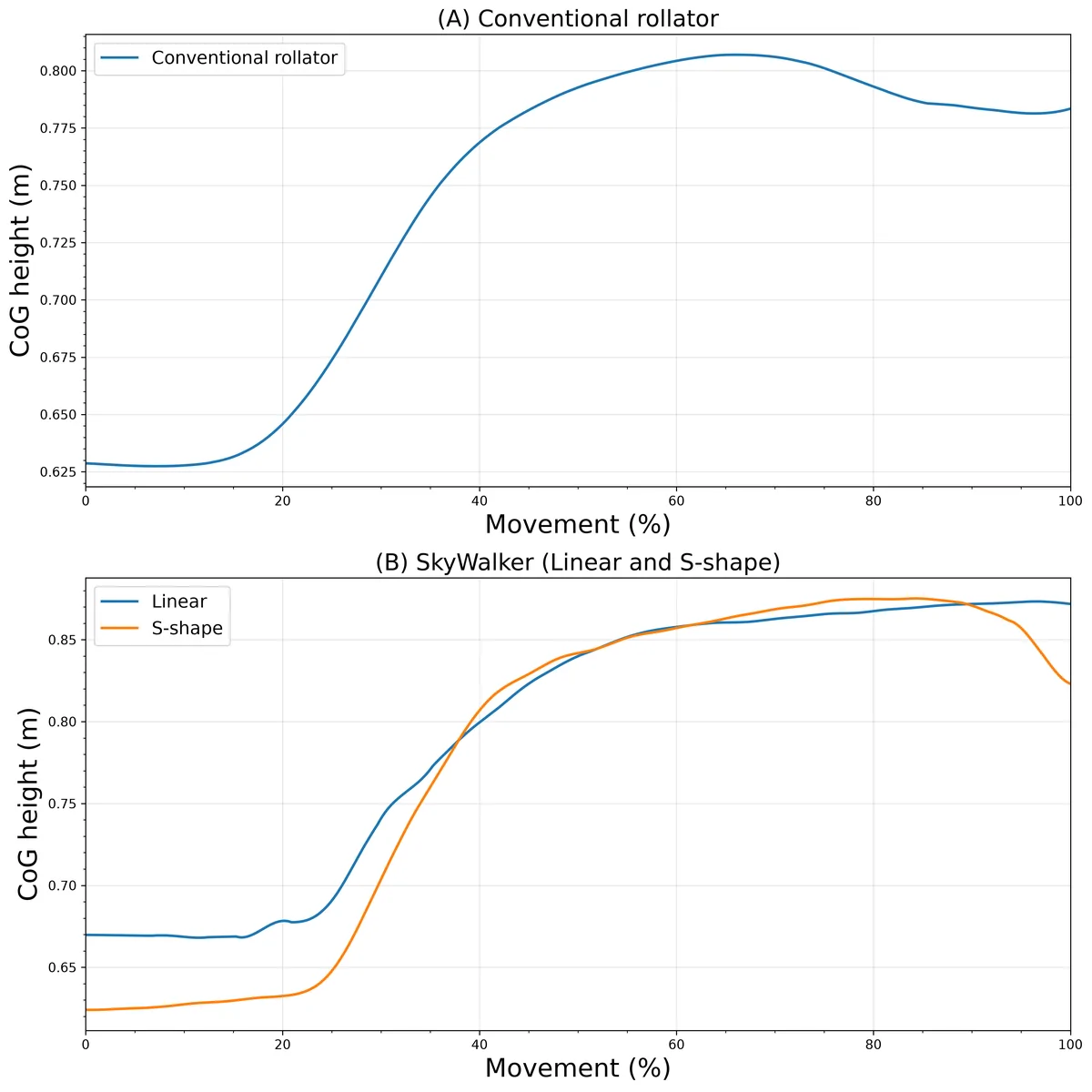
Motion-capture center of gravity height during sit-to-stand: group-mean curves (means of per-participant mean curves over successful repetitions), aligned at movement onset. (A) Conventional rollator (n=6 participants; analysis window: onset to 2 seconds). (B) SkyWalker linear (n=6) and S-shape (n=5; analysis window: 1 second before onset to 4.5 seconds). P3 is not included in the S-shape curve because the patient completed no successful S-shape repetition. The horizontal axis is the percentage of the fixed analysis window, not of each repetition’s own duration. CoG: center of gravity.

#### Kinetics

Ground-reaction forces were recorded during the SkyWalker conditions with two force plates, one under the chair and one under the participant’s feet, at 1000 Hz. The conventional-rollator condition was not instrumented with force plates. Force signals were low-pass filtered (fifth-order Butterworth) and smoothed with a 10-sample moving average. Recordings were aligned at movement onset detected from the force traces, truncated to a common analysis window, and the vertical component measured under the feet (Fz) was normalized to body weight. Per-participant condition curves are means across the repetitions retained after excluding aborted repetitions and technically unusable recordings (exact counts in the caption of Figure 3).

**Figure 3.**
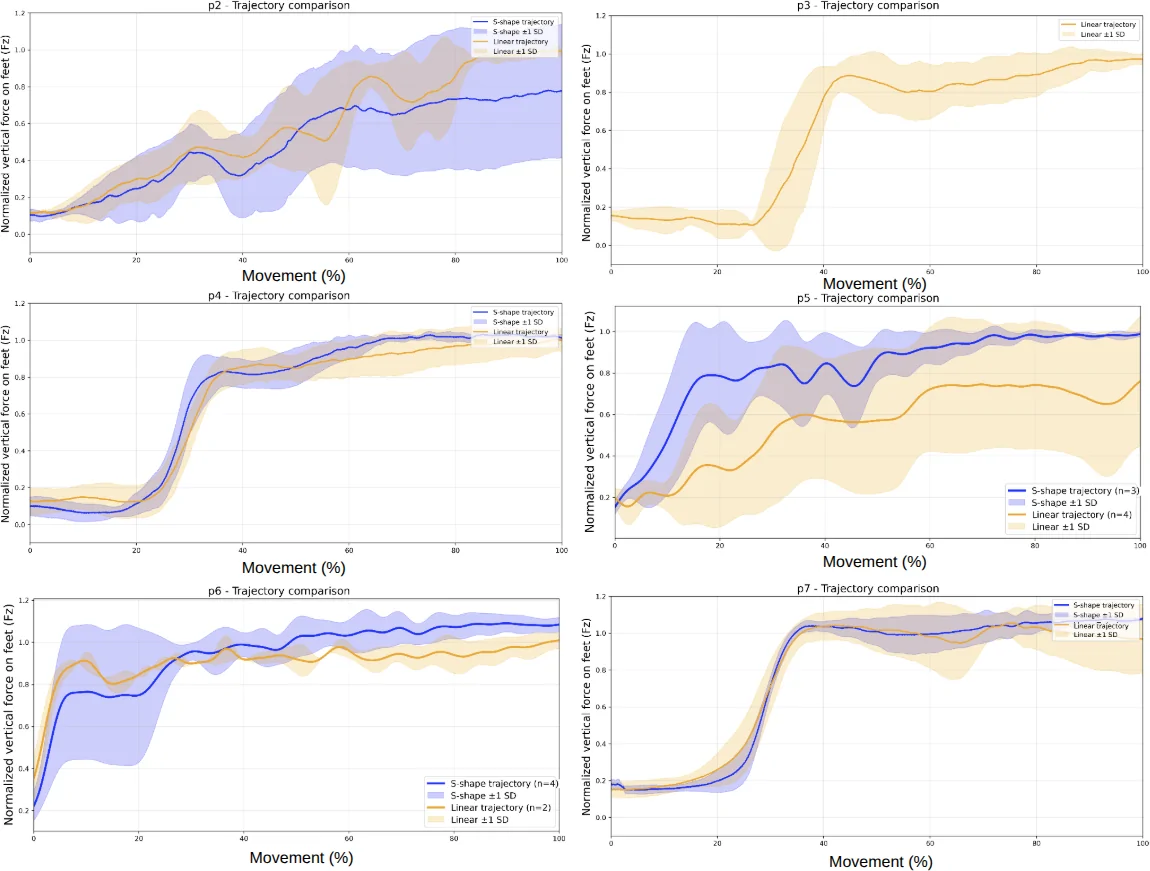
Force-plate vertical ground-reaction force measured under the feet (Fz), normalized to body weight, across the analysis window (0%‐100%) under SkyWalker assistance. Mean curves for linear and S-shape profiles are shown with shading of 1 SD for each participant (panels: P2, P3 top row; P4, P5 middle row; P6, P7 bottom row). Curves average successful repetitions with usable recordings (Linear: 4, 5, 5, 4, 2, and 4 repetitions for P2-P7, respectively; S-shape: 4, none, 4, 3, 4, and 3). No S-shape curve is shown for P3 because the patient completed no successful S-shape repetition.

### Statistical Analysis

Given the sample size (n=6) and the ordinal nature of several measures, we used exact nonparametric statistical tests, whose *P* values do not rely on large-sample approximations, and we present participant-level data and descriptive statistics as the primary results. For outcomes that involved repeated measures across the three conditions, we used the Friedman test with tie correction, with exact *P* values obtained by exhaustively permuting condition labels within participants, and tie-adjusted Kendall W reported as the effect size. Because zero (tied) paired differences are frequent at ceiling in these data and carry no directional information, pairwise condition contrasts are summarized by direction counts and analyzed with exact 2-sided sign tests (zeros reported separately), with Bonferroni correction across the 3 pairwise contrasts per outcome. Because the omnibus tests were not significant, all pairwise results are post hoc analyses and are reported as such. Paired effect estimates are given as Hodges–Lehmann pseudomedians with conservative 96.9% CIs under the symmetric-difference model, and matched rank-biserial correlations (computed after excluding zero differences) are reported descriptively. Descriptive statistics (mean, SD, median, and range, as appropriate) were calculated for baseline measures (SPPB, FES-I) and for outcome measures under each condition. Test selection was based a priori on the measurement scales and the paired design (ordinal exertion ratings; bounded success counts with substantial ceiling ties), not on pretesting for normality or homogeneity, which is uninformative at this sample size. Analyses were performed with custom enumeration scripts in Python 3.12 (NumPy/SciPy), with exact null distributions computed by exhaustive enumeration.

### Ethical Considerations

The study was approved by the Ethics Committee of the Medical Faculty Heidelberg (approval no. S-654/2019) and conducted in accordance with the Declaration of Helsinki and its later amendments. All participants provided informed consent prior to participation; participation was voluntary, and participants could withdraw at any time. Personal identifiers in figures depicting participants have been anonymized, and participants consented to the use of study photographs for publication.

## Results

### Overview

Results are reported for the 6 participants who completed the protocol (P2–P7; Table 1). Each participant attempted up to 5 STS repetitions in each within-subject condition (SkyWalker linear, SkyWalker S-shape, and conventional rollator). Because responses differed between participants, we present both participant-level outcomes (Table 2, Figures 3 and 4) and group-level summaries (Table 3).

**Table 1.** Participant demographics and baseline measures (N=6).

Participant	Age (years)	Sex	Frailty status^a^	Falls^b^	SPPB^c^	FES-I^d^	MMSE^e^
P2	70	Female	5 (Mild)	2	4	15	30
P3	84	Female	5 (Mild)	2	9	14	30
P4	82	Female	4 (Vulnerable)	2	9	8	28
P5	75	Male	4 (Vulnerable)	1	5	8	29
P6	73	Male	4 (Vulnerable)	5	6	8	28
P7	75	Female	5 (Mild)	1	3	18	27

a Frailty status: Clinical Frailty Scale (CFS) score (5=mildly frail, 4=vulnerable).

b Falls count in past year.

c SPPB: Short Physical Performance Battery (0‐12).

d Short FES-I: 7-item Short Falls Efficacy Scale–International (7-28; higher=greater concern about falling).

e MMSE: Mini-Mental State Examination (0‐30).

**Table 2.** Participant-level summary of baseline function, STS^a^ performance, perceived exertion, and acceptance (N=6).

Participant	SPPB^b^	FES-I^c^	Linear^d^ (out of 5)	S-shape^d^ (out of 5)	Rollator^d^ (out of 5)	Borg^e^	TSQ-WT^f^
P2	4	15	5	5	5	3.7	64
P3	9	14	5	0	5	3.7	67
P4	9	8	5	5	5	0.7	77
P5	5	8	4	3	5	3.3	43
P6	6	8	3	3	5	0.0	22
P7	3	18	5	5	5	1.3	69

a STS: sit-to-stand.

b SPPB: Short Physical Performance Battery.

c FES-I: Falls Efficacy Scale–International.

d Successful STS repetitions out of 5 per condition.

e Mean Borg Category-Ratio 10 scale rating across the 3 conditions.

f Telehealthcare Satisfaction Questionnaire–Wearable Technology (TSQ-WT) total score (0‐80). Domain scores are shown in Figure 4.

**Table 3.** Task performance and perceived exertion across conditions (N=6).

Condition	Success (out of 5), mean (SD)	Completed (all 5 repetitions), n (%)	Borg CR10^a^, mean (SD)
Robot			
Linear trajectory	4.50 (0.84)	4/6 (67)	1.50 (1.38)
S-shape trajectory	3.50 (1.97)	3/6 (50)	2.50 (2.43)
Conventional rollator	5.00 (0)	6/6 (100)	2.33 (1.63)

a Borg CR10: Borg Category-Ratio 10 scale.

**Figure 4.**
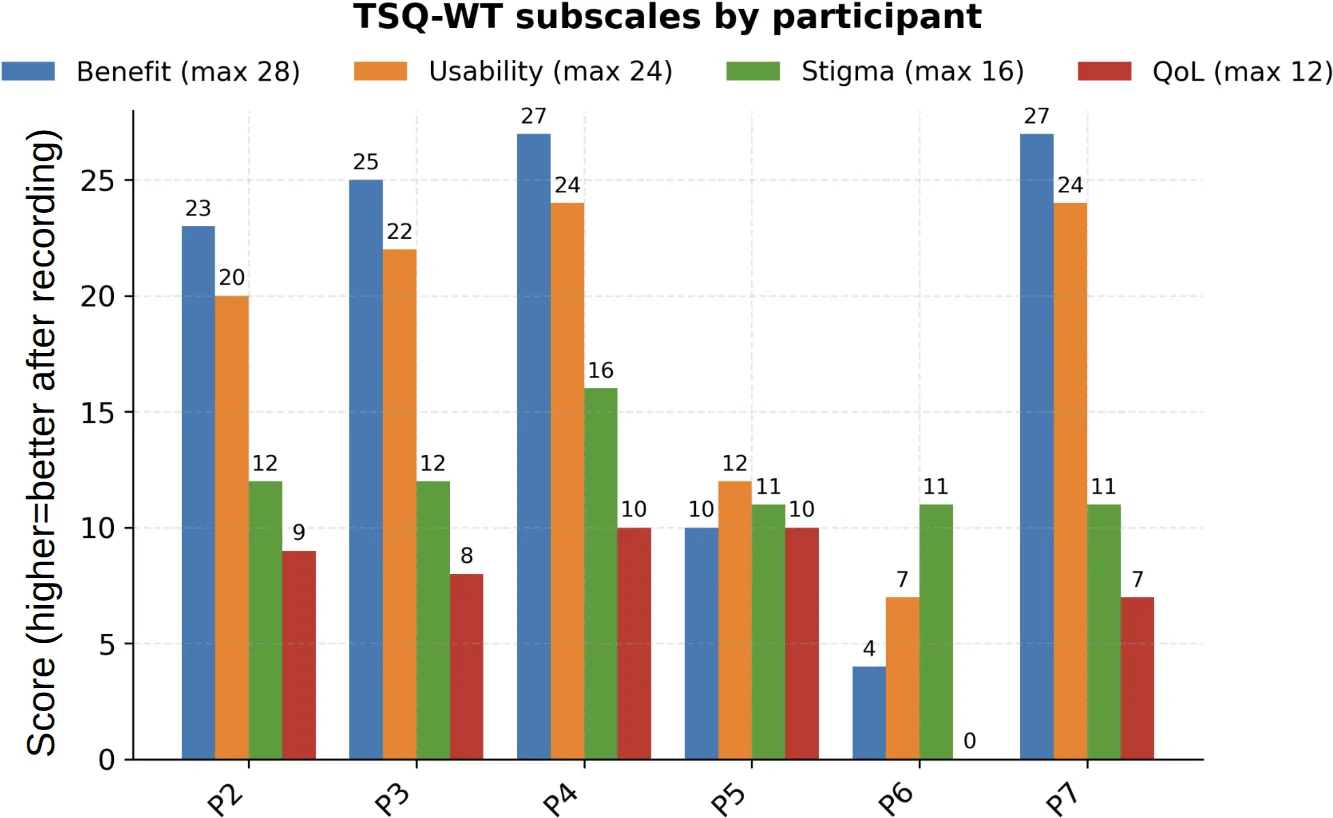
Technology acceptance (Tele-healthcare Satisfaction Questionnaire–Wearable Technology) subscales by participant (P2-P7). Bars show postrecoding subscale totals: benefit (maximum 28), usability (maximum 24), stigma (maximum 16; higher=less stigma), and quality of life (maximum 12). Items were rated 0‐4 (0=completely disagree to 4=completely agree). Negatively worded items (2, 7, 8, 11, and 15) were reverse-coded (recoded as 4 minus the raw score) so that higher scores consistently indicate more favorable responses on every item and subscale. max: maximum; QoL: quality of life; TSQ-WT: Tele-healthcare Satisfaction Questionnaire–Wearable Technology.

### Inferential Statistics

Neither perceived exertion (Friedman *χ*^2_2_^=2.1, exact *P*=.40; tie-adjusted Kendall W=0.18) nor STS success (Friedman *χ*^2_2_^=5.0, exact *P*=.11; W=0.42) differed significantly across the 3 conditions, and no pairwise comparison was significant after Bonferroni correction (Table 4; pairwise detail in Multimedia Appendix 2). Of note, with the observed tie structure the success statistic of 5.0 is the maximum attainable value, so its exact *P*n value cannot fall below .11 in this design regardless of effect size. The participant-level data below are the primary findings.

**Table 4.** Exact nonparametric analyses of the condition comparisons (N=6)^a^.

Outcome	Friedman chi-square (*df*)	Exact *P* value	Kendall W^b^
Borg CR10^c^	2.1 (2)	.40	0.18
Success (out of 5)	5 (2)	.11	0.42

a Tie-corrected Friedman statistic; the exact *P* value was obtained by exhaustive within-participant permutation of condition labels (*df* shown conventionally). With the observed tie structure, the success statistic of 5 is the maximum attainable value, so its exact *P* value cannot fall below .11 in this design.Appendix 3

b Tie-adjusted Kendall W. Pairwise contrasts (exact sign tests, direction counts, Hodges-Lehmann estimates with exact confidence intervals, and rank-biserial correlations) are post hoc analyses and are reported in Multimedia Appendix 2.

c Borg CR10: Borg Category-Ratio 10 scale.

### Feasibility and Task Performance

#### Completion and Safety Observations

Six of seven enrolled participants completed the full protocol (linear, S-shape, and rollator conditions). Across completers, no adverse events or device malfunctions were observed during STS attempts (as recorded by the study team). During P3’s 2 unsuccessful S-shape attempts, the patient was unable to complete the rise and returned to the chair under supervision without contacting the floor or sustaining injury. These events were classified as incomplete repetitions rather than falls or adverse events. Partial STS failures occurred only in SkyWalker conditions and reflected user-initiated aborts or inability to complete the full standing transition on a given repetition.

#### STS Success Across Conditions

All 6 completers achieved ceiling performance with the conventional rollator (5/5 repetitions for every participant; Table 3). With SkyWalker assistance, success was near-ceiling for most participants but showed trajectory- and participant-specific reductions. At the group level, mean success was 4.50 (SD 0.84) for linear and 3.50 (SD 1.97) for S-shape out of 5 (Table 3). At the participant level (Table 2), 3 individuals showed partial completion with SkyWalker: P3 completed no S-shape repetition (two attempts, both incomplete, after which the patient declined further S-shape attempts; 5/5 in linear), P5 completed 4/5 (linear) and 3/5 (S-shape), and P6 completed 3/5 in both robot modes. The remaining participants (P2, P4, and P7) completed 5/5 repetitions in both robot conditions. At the repetition level, 27 of 30 planned repetitions (90%) were successful under linear, 21 of 30 (70%) under S-shape, and 30 of 30 (100%) with the rollator (noting that repetitions within a participant are not independent observations). Robotic assistance therefore did not improve task success relative to the conventional rollator in this cohort. Its potential value lies in reshaping effort and movement quality rather than enabling otherwise impossible transfers.

#### Baseline Function Versus Success

Neither baseline physical function (SPPB) nor fall concern (Short FES-I) showed a monotonic descriptive relationship with the corresponding participant-level outcomes (scatter plots in Multimedia Appendix 3). Baseline physical function did not relate strictly monotonically to mean success across conditions. For example, P3 and P4 had similarly high SPPB scores (9/12) but differed in SkyWalker S-shape feasibility (P3: 0/5; P4: 5/5), while P7 had the lowest SPPB (3/12) yet completed 5/5 repetitions in all conditions (Table 2). This pattern motivates explicit case-based reporting of feasibility rather than relying on group averages alone.

### Perceived Exertion

Group-level perceived exertion differed descriptively by condition (Table 3, Figure 5). Mean Borg CR10 was lowest for SkyWalker linear (mean 1.50, SD 1.38; median 1.5, IQR 2.50), whereas SkyWalker S-shape (mean 2.50, SD 2.43; median 2.5, IQR 4.50) was comparable to the conventional rollator (mean 2.33, SD 1.63; median 2.5, IQR 2.50). At the participant level, linear was rated lower than the rollator by 3 of 6 participants, equal by 2, and higher by 1 (Figure 5, right panel). The lower mean under linear is therefore a participant-dependent pattern rather than a uniform effect, and it was specific to the linear trajectory rather than a general effect of powered support. Because exertion was rated once per condition after unequal numbers of completed repetitions, condition ratings are not perfectly workload-matched. The distribution in Figure 5 shows that low exertion values (0‐1) occurred in all conditions, but higher exertion ratings were concentrated in the S-shape condition (two participants, P3 and P5, rated it 5, “Hard”) and rollator (values up to 4).

**Figure 5.**
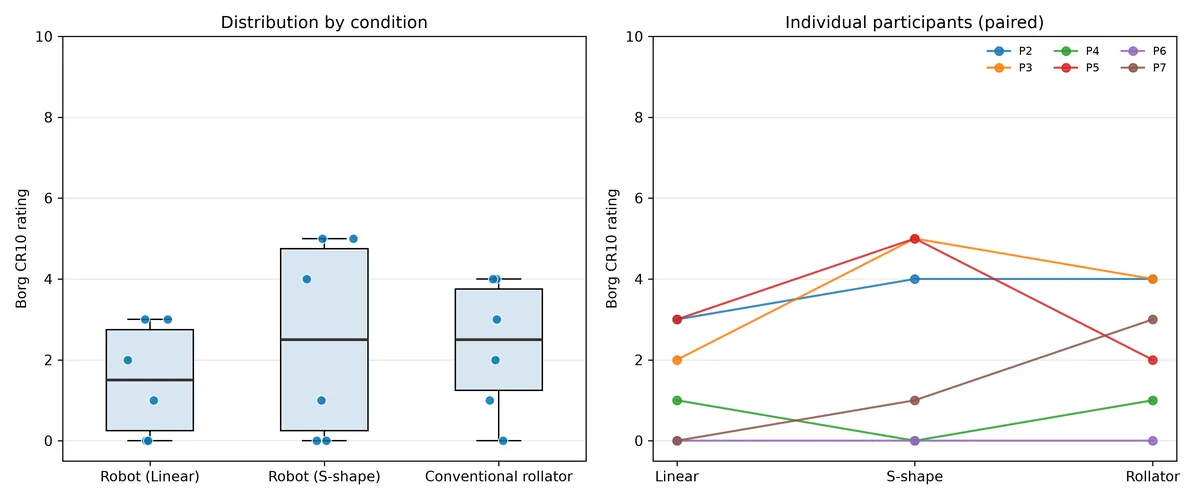
Perceived exertion (Borg CR10) by condition. Left: boxplots of postcondition exertion ratings for robot (linear), robot (S-shape), and conventional rollator, with individual participants overlaid. Right: paired participant-level ratings connected across conditions. Lower scores indicate less perceived effort.

Inter-individual variability was substantial (participant-level scatter in Multimedia Appendix 3). The relationship between fall concern (Short FES-I) and mean Borg was nonmonotonic: higher fall concern did not necessarily correspond to higher perceived exertion (eg, P7 had the highest FES-I but comparatively low mean Borg), whereas P2 and P3 reported the highest mean Borg despite only moderate-to-high FES-I scores (Table 2). The highest single-condition exertion ratings in the dataset were reported for the S-shape condition (Borg 5 by both P3, who could not complete it, and P5, who completed 3 of 5 repetitions and commented that the S-shape assistance “felt unnatural”), consistent with the larger spread seen for S-shape in Figure 5.

### Biomechanics of Assistance Profiles (CoG Kinematics and Fz Kinetics)

#### Group CoG Kinematics

Figure 2 shows the vertical CoG group-mean curves (means of the per-participant mean curves over successful repetitions), aligned at movement onset and displayed against movement percentage of each condition’s fixed analysis window. Because the rollator window is shorter than the SkyWalker window, timing should be compared within a panel rather than across panels. With the conventional rollator (Figure 2A), the CoG rose to a peak and settled slightly toward terminal stance. With SkyWalker (Figure 2B), the linear curve rose earlier within the window and more monotonically and plateaued stably, whereas the S-shape curve began its rise later within the window. Among participants with successful S-shape repetitions (n=5), the S-shape curve then overlapped the linear plateau through the mid-to-late window, with a small downward adjustment over the final portion of the window consistent with the added fore–aft component.

These kinematics align with behavioral outcomes (Table 3 and Figure 5): smoother, earlier CoG elevation in linear coincides with the highest STS success and the lowest exertion. In contrast, the S-shape’s delayed vertical commitment and its feasibility failures (which, by construction, do not appear in the successful-repetition curves) co-occur with greater variability in success and occasionally higher perceived effort for a subset. Participants with higher SPPB and smooth S-shape CoG traces generally achieved 5/5 with low exertion; those with interrupted S-shape elevation were more likely to show partial successes and higher exertion. Thus, while S-shape can be effective, its coupled forward component appears to increase coordination/balance demands during momentum transfer, which can amplify perceived effort in users with lower reserves or higher fall concern.

#### Per-Participant Kinetics

Figure 3 depicts per-participant vertical ground-reaction force measured under the feet (Fz), normalized to body weight, across the analysis window for linear and S-shape assistance (mean curves with shading of 1 SD). Linear typically produces an earlier, more progressive load transfer to the feet that stabilizes near body weight as standing is completed, with narrower SD envelopes, ie, more repeatable weight-transfer strategies across repetitions. S-shape often shows a slightly delayed Fz rise and broader variability during midcycle in several participants (eg, P5 and P6), consistent with, though not direct evidence of, an additional demand of coordinating forward translation with vertical lift. Because upper-limb and handle forces were not measured, lower foot loading cannot be uniquely attributed to device support. It may also reflect altered balance strategy or incomplete weight transfer (see Limitations section).

These kinetic signatures are descriptively consistent with the subjective and task outcomes: S-shape’s delayed and more variable Fz progression co-occurs with reduced or absent completion (0/5–3/5) and higher exertion ratings in some users, whereas participants whose linear and S-shape Fz curves overlap with narrow variability achieved 5/5 with low exertion. Rollator-condition ground-reaction forces were not recorded, so no claim is made about foot loading under assistance relative to the rollator. Likewise, because the SD envelopes summarize only the retained repetitions per curve (counts in the caption of Figure 3), they should be read as descriptive variability rather than a validated measure of movement stability or coordination.

### TSQ-WT Responses

Figure 4 summarizes TSQ-WT domain scores by participant after reverse-coding all negatively worded items so that higher scores consistently indicate more favorable evaluations. Acceptance was high for most participants: 4 of 6 scored 64 or higher of a maximum 80 (P4 77/80; P2, P3, and P7 64–69/80), with the remaining two at 43/80 and 22/80 (Table 2).

Benefit and usability were high for most participants (eg, P4 and P7 near ceiling in usability), and QoL scores were generally positive (eg, P5 QoL high within its domain range), as shown in Figure 4. One participant (P6) reported very low benefit, usability, and QoL despite a mid-range Stigma score comparable to others. These domain-level patterns support a case-based interpretation alongside feasibility and biomechanics.

### Participant-Level Patterns

Given the between-participant heterogeneity across feasibility, biomechanics, and TSQ outcomes, participant-level results are central rather than incidental. Detailed case descriptions for each participant (P2–P7), integrating baseline function, task success, exertion, Fz transfer signatures, and acceptance profiles, are provided in Multimedia Appendix 3. The recurring patterns across cases are synthesized in the Discussion. Three qualitative observations recorded by the study team merit note: P5 described the linear assistance as “unusual” and the S-shape assistance as feeling “unnatural”; P7 was observed to synchronize correctly with the S-shape assistance only from the third–fourth repetition onward, suggesting a short familiarization effect; and P3 declined to continue the S-shape condition after unsuccessful attempts.

## Discussion

### Principal Findings

This is the first study in which older adults with mobility challenges used the SkyWalker, rated it, and were instrumented while doing so. This single-site within-participant feasibility study evaluated SkyWalker with six older adult patients with mobility challenges, comparing two assist trajectories (linear and S-shape) against a conventional rollator. The principal findings are: (1) STS with the conventional rollator was at ceiling for all completers, so powered assistance did not improve task success in this cohort, and it introduced partial failures for three participants (particularly under S-shape); (2) perceived exertion was lowest under linear assistance, whereas S-shape exertion was comparable to the conventional rollator and included the two highest individual ratings; (3) biomechanically, linear produced earlier and more monotonic CoG elevation and generally more repeatable load transfer, while S-shape increased between-participant variability; and (4) responses on the unvalidated adapted TSQ-WT were heterogeneous (totals 22‐77 of 80), with 2 participants reporting low benefit/usability. One of seven enrolled participants withdrew during the robot familiarization, before attempting any study trial. In this sample, linear had a lower mean Borg rating and more completed repetitions than S-shape. These observations identify the assistance profile as a variable to prioritize in further testing, not as an established determinant of user response. Below we discuss the results explicitly RQ-by-RQ and then synthesize individual-case profiles that carry direct design and clinical implications.

### RQ1: Feasibility and Safety

RQ1 asks whether active STS assistance is feasible for older adults with mobility challenges. Feasibility of powered STS assistance was demonstrated for most participants: 6 of 7 enrolled participants completed the full protocol, 27 of 30 linear repetitions (90%) and 21 of 30 S-shape repetitions (70%) were successful, and no adverse events or device malfunctions were recorded by the study team. One enrollee withdrew during the robot familiarization (unable to stand up) before attempting any study trial, and one completer completed no S-shape repetition. Because no feasibility thresholds had been prespecified, these metrics are reported descriptively rather than as a pass/fail verdict. However, feasibility was not uniform across users or trajectories. Even among completers, SkyWalker success was not ceiling in all cases (Table 2), and S-shape showed the greatest vulnerability to partial completion (eg, P3: 0/5).

It is important to note that “feasibility” here is not equivalent to “superiority to rollator.” The rollator condition was universally successful for completers (5/5), meaning that in this cohort the value proposition of the robot is not primarily about enabling STS that is otherwise impossible; rather, it is about reshaping the perceived and biomechanical demands of the transfer. The noncompletion during early exposure is equally important evidence for feasibility: it indicates that, in a rehabilitation hospital where fatigue and confidence vary substantially, a subset of intended users may require additional familiarization, confidence-building, or trajectory personalization before they can safely and comfortably engage with powered STS support.

### RQ2: Effort Reduction and Perceived Support

RQ2 concerns perceived exertion during repeated STS. At the group level, mean Borg CR10 ratings were lowest with SkyWalker linear (lower than the rollator for 3 of 6 participants, equal for 2, higher for 1). S-shape ratings were comparable to the conventional rollator on average and included the two highest individual ratings in the dataset (Tables 3 and 4). Any effort benefit of powered assistance was therefore trajectory-specific and participant-dependent: it was observed under linear for half the participants, and not under S-shape. Because STS success with the rollator was ceiling for all completers (5/5), perceived exertion becomes a primary indicator of perceived support: the task was feasible across conditions, but it did not feel equally demanding. This is particularly relevant for adoption, as older adults may continue to complete STS with conventional aids while still experiencing high cumulative effort and fatigue across daily repetitions.

Importantly, the exertion data are not only separated by mean values but also by their dispersion. Figure 5 shows a narrower distribution for linear and the broadest spread for S-shape, whose ratings ranged from 0 to 5. This pattern supports a trajectory-specific interpretation: linear appears to provide more consistent perceived assistance across users, whereas S-shape yields more heterogeneous experiences: for some participants it remains low-effort, but for others it can feel demanding despite being “assisted.” In evaluations with the intended users, this difference in variance is often as informative as the difference in central tendency because it signals robustness (or lack thereof) across users.

We also examined whether exertion aligned with baseline fall-related concern. The relationship between Short FES-I and mean Borg was weak and non-monotonic (Multimedia Appendix 3), indicating that generalized concern about falling was not sufficient to explain who experienced the task as effortful. For example, P7 exhibited the highest FES-I score yet relatively low mean Borg, whereas P2 and P3 reported high exertion with only moderate-to-high FES-I. One plausible interpretation is that the Borg scale here captures physical effort during execution more than anticipatory concern, and that exertion rises primarily when the user must invest additional muscular or coordination effort to maintain a stable transfer under a given assistance profile. Practically, this implies that screening users only by fear of falling measures would not reliably predict perceived effort with powered assistance; evaluation and personalization should consider how the user couples their motor strategy to the device trajectory.

Finally, the effort findings are mechanically interpretable in light of the kinematics and kinetics. Linear assistance produced earlier and more monotonic CoG elevation (Figure 2) and often narrower Fz variability envelopes (Figure 3), consistent with a more repeatable weight-transfer strategy across repetitions. Such repeatability plausibly reduces the need for compensatory upper-limb pushing and stabilizing corrections, which can lower perceived exertion even when task success is unchanged. In contrast, S-shape frequently exhibited delayed/variable load transfer timing and broader mid-cycle Fz dispersion in some participants (Figure 3), consistent with a higher coordination burden during momentum transfer. This provides a coherent explanation for why S-shape can remain low-effort for some users yet produce higher exertion (and occasionally aborted repetitions) for others.

Taken together, RQ2 provides participant-dependent evidence that SkyWalker with the linear trajectory can reduce perceived effort during repeated STS, with linear appearing more robust across participants and S-shape more sensitive to individual coordination demands. From a design and deployment perspective, these findings motivate (1) treating the linear profile as the candidate default for further study, (2) brief familiarization to reduce user–device synchronization demands (directly supported by the observation that P7 synchronized with the S-shape assistance only from the third–fourth repetition), and (3) evaluation of adaptive assistance strategies in the intended population. In our prior development work [31], we implemented a vision-based adaptive trajectory controller and validated it with healthy participants; a key next step is to test such adaptive STS control with older adults with mobility challenges to determine whether real-time trajectory adaptation improves consistency of weight transfer, perceived effort, and feasibility in clinically relevant users.

### RQ3: Biomechanical Mechanisms and Trajectory-Dependent Coordination Demands

RQ3 asks how trajectory design influences STS biomechanics and whether the resulting profiles imply differences in repeatability or coordination demand. The kinematic summary in Figure 2 indicates that linear assistance is associated with earlier and more monotonic CoG elevation, whereas the S-shape profile delays vertical commitment. Among participants who completed S-shape repetitions successfully, the S-shape curve reached the same late-window plateau as linear, with a small adjustment at the end of the window. In the context of STS phase structure, these differences matter because small shifts in the timing of forward/vertical commitment can change how the user manages momentum transfer and stabilization.

The participant-level Fz results in Figure 3 add specificity: several participants exhibited narrow SD envelopes and trajectories approaching body weight late in the cycle (eg, P4 and P7), while others displayed broad SD regions and/or sustained under-loading (eg, P5; and P2 in S-shape). We interpret a broader SD envelope cautiously, as descriptive repetition-to-repetition variability over the retained repetitions rather than a validated stability metric, but in a task such as STS, where stability depends on coordinating trunk motion, foot loading, and upper-limb support, it is at least suggestive that the user is not repeating a single consistent motor strategy in that condition.

A central mechanistic implication is that S-shape likely changes the coupling between forward progression and vertical lift relative to linear. When users can accommodate this coupling, Fz rises smoothly and repeatably (eg, P4 and P7), and feasibility remains high. When users cannot, we observe either (1) noncompletion (P3 in S-shape) or (2) high variability/under-loading patterns (eg, P5), which may reflect a more cautious or inconsistent strategy under that profile. Because the current dataset does not include direct measures of upper-limb forces, attention, or balance margins, we avoid stronger causal claims; in particular, lower foot loading under assistance is compatible with several explanations (greater device support, reduced engagement, altered balance strategy, or incomplete transfer) that cannot be separated without handle-force sensing. Nevertheless, the combined CoG timing differences and the participant-level Fz variability are descriptively consistent with linear being the better-tolerated trajectory in this sample, while S-shape can be effective for some users but appears more sensitive to individual capacity; coordination demand itself was not measured.

### RQ4: Acceptance, Stigma, and Quality-of-Life Relevance

RQ4 concerns user experience as measured by TSQ-WT domains (benefit, usability, self-concept/stigma, and quality of lQoL). Overall acceptance was high for most participants (4 of 6 at 64 or higher of a maximum 80), with domain-level differences across the cohort (Figure 4). Several participants reported high benefit and usability with low stigma after reverse-coding (eg, P4 and P7), suggesting that powered STS assistance can be perceived as both useful and acceptable from a self-concept perspective in this cohort. Because stigma and self-concept concerns can be important barriers to adoption of assistive technologies in older adults, it is notable that stigma was not a dominant negative factor for most participants in this study.

The key nuance is dissociation across domains. P6 reported very low benefit and usability while having a mid-range Stigma score comparable to others (Figure 4), implying that low acceptance can stem from perceived lack of utility/usability even when stigma is not high. Conversely, P3 reported high Benefit and Usability despite reduced S-shape feasibility (Table 2), suggesting that some users may recognize the potential value of the system even if one control profile does not yet fit their motor strategy. For design and deployment, this means that acceptance should not be treated as a single scalar; rather, domain-level patterns can help distinguish “the device feels stigmatizing” from “the device does not help me” from “the device might help me but needs tuning/training.” Because the TSQ-WT was administered once after exposure to both robotic trajectories, these acceptance ratings reflect the system as a whole and cannot be attributed to a specific trajectory. Trajectory-specific acceptance measurement is an important refinement for future studies.

### Individual Differences and Case-Based Interpretation

#### Overview

The cohort was heterogeneous enough that individual differences are themselves a result. We therefore use a case-based lens and summarize recurring case patterns across feasibility, biomechanics, and acceptance, with direct design and deployment implications (full case narratives in Multimedia Appendix 3):

#### Case Pattern 1: Robust Users with Consistent Biomechanics and High Acceptance (P4, P7)

These participants showed ceiling feasibility across conditions, smooth and repeatable Fz transfers (narrow SD envelopes and loading near body weight late in the cycle), and high TSQ benefit/usability with low Stigma (Figures 3 and 4). Notably, this pattern includes both high-function/low-concern (P4) and low-function/high-concern (P7) participants (Table 2), indicating that baseline function alone does not determine successful adoption; stable coupling between user strategy and assistance profile appears critical. An unmeasured but plausible mediator is upper-limb capacity: 3 of 6 completers (P2, P5, and P7) could not rise once without arm use in the SPPB chair-stand test, yet completed assisted repetitions by loading the handles. This observation shows that arm support was necessary for these users. Whether upper-limb strength, joint range of motion, or the ability to time arm loading with the moving handles mediates success with handle-based assistance is a hypothesis this dataset cannot test, and direct upper-limb measurements are needed.

#### Case Pattern 2: Feasible but Biomechanically Trajectory-Sensitive Users (P2)

P2 achieved ceiling success in all conditions yet displayed a marked trajectory separation in Fz (S-shape remaining under body weight late in the movement with larger variability) and high mean Borg across conditions (Figure 3). This suggests that feasibility alone can mask important differences in how assistance is being used: a user may complete every repetition while still experiencing higher effort or relying on a less repeatable strategy under one profile. These cases motivate monitoring transfer quality rather than success/failure alone.

#### Case Pattern 3: Users with Reduced Feasibility or Low Perceived Benefit/Usability (P5, P6, and the Noncompletion Case)

P5 and P6 showed partial completion with SkyWalker, broad Fz variability, and comparatively lower acceptance (especially P6 with very low benefit/usability) (Table 2, Figures 3 and 4). P5’s remarks that the assistance felt “unusual”/“unnatural” put words to this pattern. The noncompletion during early exposure underscores that a subset of intended users may be unable to use the system at all; the documented circumstances are reported in the participants section. Together, these cases suggest that a one-size-fits-all trajectory may be insufficient for clinical deployment.

Across patterns, acceptance can remain high even when one trajectory is not feasible (P3), supporting the interpretation that trajectory adaptation and/or additional familiarization may improve usability for some users without reducing perceived benefit.

### Limitations and Future Work

This study evaluated six completers in a single session, which restricts statistical power and the ability to characterize learning effects. The sample size is largely structural: recruiting and testing patients in a rehabilitation hospital requires aligning with clinical schedules, medical status, fatigue tolerance, and safety considerations, and it is difficult to access larger cohorts with uniform diagnoses or impairment profiles within the same time window. As a result, the cohort is clinically heterogeneous, which reflects the intended user group in a clinical setting but constrains between-participant comparability. We note that this remains a supervised, instrumented, single-session experiment. It does not establish real-world or unsupervised use, and the comparator rollator differs from the SkyWalker in geometry, handle configuration, and permitted support strategy, so condition differences cannot be attributed to actuation alone.

The rollator condition exhibited ceiling success, which limits the interpretability of success-rate comparisons and shifts attention to effort and biomechanical quality. Additional limitations concern the measurements themselves. First, upper-limb and handle forces were not recorded, so reduced foot loading under assistance cannot be uniquely attributed to device support. Second, although the TSQ-WT has previously been used to evaluate STS assistance provided by a smart walker in older adults [22], a formal comprehensive evaluation of its psychometric properties is still lacking, which limits the interpretability of the questionnaire-derived findings. In addition, it was administered once for the robotic system as a whole, so acceptance cannot be linked to a specific trajectory. Third, biomechanical group curves average repetitions aligned at movement onset over a fixed time window rather than normalizing each repetition to its own movement duration. Per-curve repetition counts are disclosed in the figure captions. Fourth, seat height was adjusted to each participant’s body height rather than fixed across participants, which affects raw between-participant comparisons of vertical CoG height. Fifth, although condition order was randomized, a short familiarization/learning component was observed for one participant, so within-session learning effects cannot be excluded. Future work should therefore include larger samples, multi-session exposure to capture learning/adaptation, direct comparative satisfaction measures across conditions, trajectory-specific acceptance measurement, and additional sensing (eg, upper-limb load sharing) to more directly explain why some participants exhibit sustained under-loading or high variability in Fz under specific trajectories.

### Conclusions

This study is the first evaluation of SkyWalker with its intended users. It evaluated the feasibility, perceived effort, biomechanics, and user experience of SkyWalker, a rollator providing powered STS assistance, in older adults with mobility challenges, using a within-subject comparison of two assistance trajectories (linear and S-shape) against a conventional rollator. Addressing RQ1, SkyWalker STS assistance was feasible for most enrolled users who completed testing (6/7), with no observed adverse events among completers; however, feasibility was not uniform, as one participant was unable to complete testing and several participants showed partial completion in at least one powered condition, particularly with the S-shape trajectory. Robotic assistance did not improve task success relative to the conventional rollator. Addressing RQ2, perceived exertion (Borg CR10) was lowest with the linear trajectory, whereas the S-shape trajectory offered no exertion advantage over the conventional rollator. The effort benefit of powered assistance was therefore trajectory-specific rather than general. Addressing RQ3, the assistance profiles produced distinct biomechanical signatures: linear was associated with earlier and more monotonic CoG elevation, while participant-level Fz patterns revealed substantial inter-individual variability in load transfer timing and repeatability, with several users exhibiting broader variability or sustained under-loading in specific trajectories. These findings support the interpretation that trajectory design influences coordination constraints and that robust assistance should prioritize repeatable, stable weight transfer rather than success alone. Addressing RQ4, responses on the unvalidated adapted TSQ-WT were heterogeneous (22‐77 of 80): benefit, usability, and self-concept ratings were high for 4 participants but low for 2, and these descriptive totals do not establish validated technology acceptance, including one markedly low-acceptance profile.

Overall, the results suggest that SkyWalker STS assistance with a simple, monotonic trajectory can reduce perceived effort for a subset of users and was rated positively by most, while trajectory-specific and participant-specific feasibility constraints point to personalization, training, and adaptive trajectory policies. In particular, the linear trajectory is the profile to prioritize in further testing in this population, whereas S-shape may be effective for some users but appears more sensitive to individual capacity. These observations come from a within-participant feasibility study with six completers and are reported as feasibility evidence, not as efficacy claims. The results are already being used to design the next generation of the SkyWalker, which addresses the challenges identified here. Future work should evaluate multi-session learning effects, include direct comparative satisfaction/preference measures against conventional aids, and incorporate larger samples with stratification by functional status to determine which users benefit most and how assistance trajectories should be tuned for safe, reliable clinical and home deployment.

## Supplementary material

10.2196/101303 Multimedia Appendix 1 Tele-healthcare Satisfaction Questionnaire.

10.2196/101303 Multimedia Appendix 2 Pairwise analyses.

10.2196/101303 Multimedia Appendix 3 Case descriptions.

## Abbreviations

Borg CR10: Borg Category-Ratio 10 scale
CFS: Clinical Frailty Scale
CoG: center of gravity
CoM: center of mass
FES-I: Falls Efficacy Scale–International
Fz: vertical ground-reaction force
MMSE: Mini-Mental State Examination
QoL: quality of life
RQ: research question
SPPB: Short Physical Performance Battery
STS: sit-to-stand
TSQ-WT: Tele-healthcare Satisfaction Questionnaire–Wearable Technology
