# Supplementary material for: Robotic Rollator Assistance for Sit-to-Stand Transfers in Older Adults with Mobility Challenges: Within-Participant Feasibility Study
Source: JMIR Rehabil Assist Technol. 2026 Nov 11;13:e101303. doi: 10.2196/101303 (PMC13754262; doi:10.2196/101303)
Supplement: Supplementary file 1 [file rehab-v13-e101303-s001.docx]

# Multimedia Appendix 1: TSQ-WT: adapted 20-item instrument and domain assignments

Scale for every item: 0 = not at all; 1 = mostly not; 2 = partly; 3 = mostly; 4 = completely. Items marked * are negatively worded and were reverse-coded (recoded as 4 minus the raw score). Domain assignments follow the study’s adapted scoring: Benefit (7 items, maximum 28), Usability (6 items, maximum 24), Self-concept/Stigma (4 items, maximum 16; higher = less stigma), and Quality of Life (3 items, maximum 12).

| # | Domain | English item |
| --- | --- | --- |
| 1 | Benefit | I can benefit from the SkyWalker’s stand-up aid |
| 2 | Usability* | Using it requires effort |
| 3 | Usability | Using it is an interesting challenge |
| 4 | Benefit | Could improve my physical well-being |
| 5 | Benefit | The effort is worthwhile for me |
| 6 | Usability | Functions reliably |
| 7 | Stigma* | Reminds me of loss of independence |
| 8 | Stigma* | Causes unpleasant feelings |
| 9 | Benefit | I get maximum benefit from it |
| 10 | Usability | Is easy to use |
| 11 | Stigma* | Makes me feel older than I am |
| 12 | Quality of Life | Could promote my social contacts |
| 13 | Benefit | Helps me achieve my goals |
| 14 | Quality of Life | I feel safe using it |
| 15 | Stigma* | Would be embarrassing to use in front of others |
| 16 | Quality of Life | Helps maintain my independence |
| 17 | Benefit | Would recommend to others |
| 18 | Usability | I feel comfortable using it |
| 19 | Usability | I like using such technologies |
| 20 | Benefit | Has positive effect on me |

Domain assignments (after reverse-coding of items 2, 7, 8, 11, and 15): Benefit = items 1, 4, 5, 9, 13, 17, 20 (0–28); Usability = items 2*, 3, 6, 10, 18, 19 (0–24); Self-concept/Stigma = items 7*, 8*, 11*, 15* (0–16); Quality of Life = items 12, 14, 16 (0–12). Total score = sum of all 20 items (0–80).

The questionnaire was administered in German, using the German-language version of the TSQ-WT that was already available from the instrument’s developers and originated from the EU project in which the TSQ-WT was developed (Chiari et al, 2009); no translation was performed within the present study. The English wordings above are translations of the administered items, provided for reporting.
