# Supplementary material for: Robotic Rollator Assistance for Sit-to-Stand Transfers in Older Adults with Mobility Challenges: Within-Participant Feasibility Study
Source: JMIR Rehabil Assist Technol. 2026 Nov 11;13:e101303. doi: 10.2196/101303 (PMC13754262; doi:10.2196/101303)
Supplement: Supplementary file 2 [file rehab-v13-e101303-s002.docx]

# Multimedia Appendix 3: Post hoc pairwise analyses

All pairwise analyses are post hoc (the omnibus Friedman tests in Table 4 of the main text were not significant). Differences are first condition minus second; higher Borg indicates greater exertion, higher success is better. Direction counts give the number of participants with higher/lower/tied values (n+/n−/n0). Sign-test P values are exact and two-sided, conditioned on nonzero differences (ties remain visible in n0). Adjusted P values apply Bonferroni correction across the three pairwise contrasts per outcome. Hodges–Lehmann (HL) values are pseudomedians of the paired differences with conservative 96.9% exact confidence intervals under the symmetric-difference model (not multiplicity-adjusted). r is the matched rank-biserial correlation computed after excluding zero differences (descriptive; magnitudes are inflated when few pairs are nonzero).

| Outcome | Contrast | n+/n−/n0 | HL (96.9% CI) | r | Exact P | Adjusted P |
| --- | --- | --- | --- | --- | --- | --- |
| Borg CR10 | Linear vs S-shape | 1/4/1 | −1.0 (−3.0 to 1.0) | −0.73 | .38 | >.99 |
| Borg CR10 | Linear vs Rollator | 1/3/2 | −1.0 (−3.0 to 1.0) | −0.70 | .63 | >.99 |
| Borg CR10 | S-shape vs Rollator | 2/2/2 | 0.0 (−2.0 to 3.0) | 0.10 | >.99 | >.99 |
| Success (of 5) | Linear vs S-shape | 2/0/4 | 0.5 (0.0 to 5.0) | 1.00 | .50 | >.99 |
| Success (of 5) | Linear vs Rollator | 0/2/4 | −0.5 (−2.0 to 0.0) | −1.00 | .50 | >.99 |
| Success (of 5) | S-shape vs Rollator | 0/3/3 | −1.0 (−5.0 to 0.0) | −1.00 | .25 | .75 |

Success values use the P3 S-shape count of 0 of 5.
