# Supplementary material for: Robotic Rollator Assistance for Sit-to-Stand Transfers in Older Adults with Mobility Challenges: Within-Participant Feasibility Study
Source: JMIR Rehabil Assist Technol. 2026 Nov 11;13:e101303. doi: 10.2196/101303 (PMC13754262; doi:10.2196/101303)
Supplement: Supplementary file 3 [file rehab-v13-e101303-s003.docx]

# Multimedia Appendix 2: Individual case descriptions (P2–P7)

## P2

P2 (woman, 70 years) had low physical function (SPPB 4/12) and elevated fall concern relative to several peers (Short FES-I 15). Task feasibility was high: P2 completed 5/5 repetitions in all three conditions. Despite this ceiling success, P2 reported the highest mean Borg across conditions (3.7; Linear 3, S-shape 4, rollator 4). Biomechanically, P2 showed a clear separation between trajectories: Linear Fz progressed to near-body-weight loading late in the cycle, whereas S-shape remained substantially lower through late movement with a broader SD envelope. Acceptance was positive (TSQ-WT total 64/80), with mid-to-high Benefit and Usability and low Stigma after reverse-coding.

## P3

P3 (woman, 84 years) demonstrated high baseline physical function (SPPB 9/12) with moderate-to-high fall concern (FES-I 14). Feasibility differed sharply by trajectory: P3 completed 5/5 repetitions in Linear but no S-shape repetition (0/5): the patient attempted the S-shape condition twice, could not complete the rise either time, returned to the chair under supervision without contacting the floor or sustaining injury, and declined further S-shape attempts. The patient nonetheless provided the joint-highest single-condition exertion rating in the dataset for S-shape (Borg 5). In the kinetics figure, the P3 panel shows only the Linear trajectory because the patient completed no successful S-shape repetition; the Linear curve rises to near-body-weight loading by late movement but with visible mid-cycle variability. Despite the S-shape feasibility failure, P3 reported high acceptance (TSQ-WT total 67/80) with strong Benefit and Usability scores.

## P4

P4 (woman, 82 years) had high baseline physical function (SPPB 9/12) and the lowest fall concern in the cohort (FES-I 8). Feasibility was ceiling across conditions (5/5 in all three). P4 reported low Borg ratings (Linear 1, S-shape 0, rollator 1) and showed highly consistent Fz patterns: both trajectories displayed a rapid rise to near-body-weight loading with relatively narrow SD envelopes. P4 reported the highest acceptance overall (TSQ-WT total 77/80), with near-ceiling Usability and the maximum Stigma score after reverse-coding. The study log also notes that P4 did not perceive a speed difference between the two robot trajectories.

## P5

P5 (man, 75 years) had moderate baseline function (SPPB 5/12; unable to rise once without arm use) and low fall concern (FES-I 8). Feasibility was reduced for both robot trajectories (4/5 Linear; 3/5 S-shape) while the rollator remained 5/5. P5’s exertion ratings were the second-highest in the cohort (mean 3.3: Linear 3, S-shape 5, rollator 2), and the study team recorded that the patient found the Linear assistance “unusual” and that the S-shape assistance “felt unnatural”. P5 exhibited sustained under-loading in both trajectories: mean Fz remained below body weight through late movement with broad SD envelopes. Acceptance was mixed (TSQ-WT total 43/80): Benefit and Usability were comparatively low, while Quality of Life was high within its domain range.

## P6

P6 (man, 73 years) had moderate baseline function (SPPB 6/12) and low fall concern (FES-I 8). Feasibility was reduced for both robot trajectories (3/5 Linear; 3/5 S-shape) while the rollator was 5/5. P6 reported the lowest exertion across all conditions (Borg 0 everywhere). Kinetically, P6 showed trajectory-dependent variability: both trajectories approached near-body-weight loading by late movement, but SD envelopes were broad in S-shape during the early-to-mid cycle and a distinct mid-cycle dip with a broad SD region appeared in Linear. Acceptance was the lowest in the sample (TSQ-WT total 22/80), driven by very low Benefit and Usability despite a mid-range Stigma score after reverse-coding.

## P7

P7 (woman, 75 years) had the lowest baseline physical function (SPPB 3/12; unable to rise once without arm use) and the highest fall concern (FES-I 18). Despite this, feasibility was ceiling across all three conditions (5/5 each). P7’s exertion ratings were comparatively low (Linear 0, S-shape 1, rollator 3), illustrating that higher fall concern did not correspond to higher perceived exertion in this dataset. The study team noted that P7 synchronized correctly with the S-shape assistance only from the third–fourth repetition onward, consistent with a short familiarization effect. Both trajectories showed a smooth rise to near-body-weight loading early in the cycle; Linear displayed a broader SD region in mid-to-late movement than S-shape. Acceptance was high (TSQ-WT total 69/80) with near-ceiling Usability and low Stigma after reverse-coding.

## Participant-level scatter plots

Neither baseline measure showed a monotonic descriptive relationship with the corresponding outcome (e.g., P7 had the lowest SPPB and highest FES-I yet ceiling success with low exertion).

SPPB total score (0–12; higher = better lower-extremity function) versus mean STS success proportion across the three conditions, labeled by participant.

Short FES-I (7–28; higher = more concern about falling) versus mean Borg CR10 across the three conditions, labeled by participant.
